# Expression, purification, crystallization and preliminary X-ray diffraction analysis of swine leukocyte antigen 2 complexed with a CTL epitope AS64 derived from Asia1 serotype of foot-and-mouth disease virus

**DOI:** 10.1186/s12917-018-1742-3

**Published:** 2018-12-18

**Authors:** Feng-shan Gao, Lei Feng, Ping Jiang, Zi-bin Li, Hua Gao, Xiao-xin Zhai, Zong-hui Zhang, Xiao Hu

**Affiliations:** 1 Department of Bioengineering, |College of Life Science and Technology, Dalian University, Xuefu street 10, Dalian, Liaoning 116622 People’s Republic of China; 2 Department of Microbiology and Immunology, College of Veterinary Medicine, China Agricultural University, Beijing, 100094 People’s Republic of China

**Keywords:** Swine leukocyte antigen 2, Crystal structure, Foot-and-mouth disease virus, Asia 1 serotype

## Abstract

**Background:**

Currently, the structural characteristics of the swine major histocompatibility complex (MHC) class I molecule, also named swine leukocyte antigen class I (SLA-I) molecule need to be further clarified.

**Results:**

A complex of SLA-I constituted by an SLA-2*HB01 molecule with swine β_2_-microglobulin and a cytotoxic T lymphocyte (CTL) epitope FMDV-AS64 (ALLRSATYY) derived from VP1 protein (residues 64–72) of Asia 1 serotype of foot-and-mouth disease virus (FMDV) was expressed, refolded, purified and crystallized. By preliminary X-ray diffraction analysis, it was shown that the diffraction resolution of the crystal was 2.4 Å and the space group belonged to P2_1_2_1_2_1_ with unit cell parameters a = 48.37, b = 97.75, c = 166.163 Å.

**Conclusion:**

This research will be in favor of illuminating the structural characteristics of an SLA-2 molecule associated with a CTL epitope derived from Asia1 serotype of FMDV.

## Background

Class I of major histocompatibility complex (MHC) molecules are membrane-surface proteins, which are mainly responsible for binding and presenting endogenous antigenic peptides degraded from proteins coded by virus genome in target cells [1] or autoantigen [2]. These endogenous antigenic peptides usually constituted by 8–10 residues in length are derived from endogenous antigens degraded by cellular proteasome [3, 4]. When a peptide is complexed and correctly refolded with MHC class I molecule in the antigen presenting cells (APCs), it will be presented to the membrane surface of the APCs to intrigue the cytotoxic T lymphocytes (CTLs). On this occasion, the peptide is defined as a CTL epitope [5]. Once CTLs are activated by the CTL epitopes, CTLs will recognize the epitopes and then kill the infected cells [6]. Therefore, the expressed MHC class I molecules on membrane of APCs should contain three components: a polymorphic heavy chain (α chain) of class I, a monomorphic light chain of β_2_-microglobulin (β_2_m) noncovalently linking the α3 domain of the heavy chain and an epitope bound in a groove formed by the α1 and α2 domains of the heavy chain [7]. Analysis of structural characteristics of MHC class I is necessary so that the mechanism of antigen presentation associated with MHC class I molecules will be explored.

MHC class I genes in pigs (*Sus scrofa* domestica) located in the 7p1.1 band of the short arm of chromosome 7 are also named as swine leukocyte antigen class I (SLA-I) [8]. There are three constitutively expressed classical and polymorphic SLA-I genes in the genome, namely SLA-1, SLA-2 and SLA-3 [9]. Among them, SLA-2 is different from SLA-1 and SLA-3 in the N-terminal of their coding regions as previously described [10]. The swine β_2_m (sβ_2_m) is monomorphic and noncovalently links with the heavy chain of the SLA-I molecules, which bind a viral or an auto CTL epitope. Foot-and-mouth disease virus (FMDV) is a great danger to cloven-hoofed animals including pigs, because it can cause animals to develop an acute, febrile, and highly contagious infectious disease [11]. In FMDV, there are seven serotypes named as A, O, C, Asia1, SAT1, SAT2, and SAT3. However, none of them have mutual cross-immunity [12, 13]. Among them, the Asia1 serotype often occurs in Asian countries as previously reported [14, 15]. Therefore, to further epitope vaccine development, more CTL epitopes and their interactions with SLA-I should be investigated. Recently, crystal data of the SLA-1, SLA-2 and SLA-3 had been announced, and a few CTL epitopes derived from swine-origin influenza virus, O serotype of FMDV, Ebola virus and respiratory syndrome virus (PRRSV) were also discovered [16–19]. However, crystal of SLA-2 associated with CTL epitope derived from Asia1 serotype of FMDV remains elusive.

In this article, we introduce the expression, refolding, purification, crystallization and preliminary X-ray diffraction analysis of SLA-2*HB01 with an AS64 CTL epitope derived from the Aisa1 serotype of FMDV.

## Methods

### Expression and isolation of the proteins of SLA-2*HB01 and sβ_2_m

To construct the expression system of the SLA-2 haplotype HB01 allele (GenBank accession No. AB602431) coding for 275 amino acids in extracellular domain, a pair of primers was designed as shown in Table 1. The PCR product was recovered and cloned into pMD®19-T simple vector as previously described [10, 19]. After identification by digestion with *Nde* I and *Xho* I and followed by sequencing, the interest of SLA-2*HB01 was further cloned into the pET21a (+) vector. The recombinant pET21a (+) containing the sβ_2_m had been done in our laboratory previously [19]. The two recombinant SLA-2*HB01 and sβ_2_m plasmids were induced to express in BL21 (Rosetta) *Escherichia coli* strain. The inclusion bodies were extracted as follows [17, 19]: In 2 L Luria–Bertani medium (LB), the SLA-2*HB01 and sβ_2_m expression strains were inoculated and incubated at 180 rpm for 3–4 h in a shaking incubator at 37 °C until the OD_600_ value reached 0.5–0.6. Then, a final concentration of 1 mM isopropyl-β-D-thiogalactopyranoside (IPTG) was used in the medium to induce the interest of proteins to express in same cultivating environment as above. After 5 h, the bacteria were collected and cooled at 4 °C for 30 min. Then the bacteria were centrifugated for 15 min at 6000 rpm at 4 °C to collect pellets followed by washing them for three times with a solution buffer consisting of 50 mM Tris–HCl, 100 mM NaCl, 10 mM EDTA, 0.5% (*v*/v) Triton X-100, 1 mM DTT. In the end, a guanidinium chloride buffer [6 M guanidinium chloride, 100 mM NaCl, 10 mM EDTA, 50 mM Tris–HCl pH 8.0, 10% (v/v) glycerine, 10 mM DTT] was used to dissolve the isolated inclusion body proteins to a final concentration of 30 mg/mL.

**Table 1:** SLA-2*HB01-AS64-sβ_2_m complex information

Source organism	*Sus scrofa*
DNA source	Plasmid DNA
Upper primer	GGAATTCCATATGGGCCCGCATTCCCTGAGCTATTCTTAC
Lower primer	AGTCTCGAGTTAGTCCCATCTCAGGGTGAGGGGCTCCTGC
Cloning vector	pMD®19-T simple
Expression vector	pET21a (+)
Expression host	*E. coli* (Rosetta)
Complete amino-acid sequence of the construct produced	GPHSLSYSYTAVSRPDRGDSRFFIVGYVDDTQFVRFDSDAPNAKMEPRAQWIQQEGQEYWDRETQISKETAQNYRVDLNTLRGYYNQSEAGSRTIQRVYGCYLGPDGLLLRGYRQDAYDGADYIALNEDLRSWTAADMAAQITKRKWEVVNEAEGERSYLQGRCVEWLQKYLVMGKDTLQRAEPPKTHVTRHPSSDLGVTLRCWALGFYPKEISLSWQREGQDQSQDMELVETRPSGDGTFQKWAALVVPPGEEQSYTCHVQHEGLQEPLTL RW**ALLRSATYY***VARPPKVQVYSRHPAENGKPNYLNCYVSGFHPPQIEIDLLKNGEKMNAEQSDLSFSKDWSFYLLVHTEFTPNAVDQYSCRVKHVTLDKPKIVKWDRDH*

The restriction-enzyme cleavage sites are underlined in the upper and lower primers. The normal font, bold and italics of residues expressed by the vector are from the heavy chain of SLA-2*HB01, AS64 and the light chain of sβ_2_m in the amino-acid sequences, respectively

### Refolding and purification of the SLA-2*HB01-AS64-sβ_2_m complex

The refolded SLA-I (SLA-2*HB01-AS64-sβ_2_m) complex was carried out as described previously [6] with improvement recommended by Feng et al. [19]. Firstly, the epitope AS64 (ALLRSATYY) derived from the VP1 protein residues 64–72 of Asia1 FMDV was firstly dissolved in dimethyl sulfoxide (DMSO) and then diluted in water. The inclusion body proteins of SLA-2*HB01 and sβ_2_m were renatured and refolded with the AS64 epitope according to a 1:1:3 molar ratio by using the gradual dilution method in a refolding buffer (100 mM Tris pH 8.0, 400 mM L-Arg HCl, 2 mM EDTA, 5 mM GSH, 0.5 mM GSSH, 0.5 mM PMSF) at 4 °C. After refolding for 24 h, the soluble SLA-2*HB01-AS64-sβ_2_m complex was further concentrated and purified by chromatography separation on a Superdex 200 16/60 HiLoad size-exclusion column (GE Healthcare) followed by an anion-exchange chromatography Resource Q (GE Healthcare). SLA-2*HB01-AS64-sβ_2_m complex information is shown in Table 1.

### Crystallizing the SLA-2*HB01-AS64-sβ_2_m complex

The final purified SLA-2*HB01-AS64-sβ_2_m complex proteins (~ 44 kDa) were further dialyzed against a crystallizing buffer (50 mM NaCl, 20 mM Tris–HCl pH 8.0) overnight at 4 °C and then concentrated to 15 mg/mL. Crystallizing assays were tried with Index and Crystal Screen kit (Hampton Research) at 4 °C and 18 °C by using the sitting-drop vapor diffusion method as previously described [19, 20]. Two protein solution drops each with 1 μl (at 7.5 and 15 mg/mL) and 1 μL of reservoir crystallizing buffer were placed and mixed together over a well containing 120 μL reservoir solution using an MRC Maxi plate (HR3–179, Hampton Research). Crystals of SLA-2*HB01-AS64-sβ_2_m were obtained at 14th day by using a protein concentration of 15 mg/mL in the Index solution No. 79 (0.2 M Ammonium acetate, 25% *w*/*v* PEG 3350, 0.1 M BIS-TRIS pH 6.5) at 4 °C. Crystallizing condition is shown in Table 2.

**Table 2:** Crystallizing condition

Method	Sitting-drop vapor diffusion
Temperature (°C)	4
Protein concentration (mg/mL)	15
Buffer composition of protein solution	50 mM NaCl, 20 mM Tris–HCl pH 8.0
Composition of reservoir solution	0.1 M BIS-TRIS pH 6.5, 0.2 M Ammonium acetate, 25% (*w*/*v*) PEG 3350
Volume and ratio of drop	1 μL protein solution mixed with 1 μL reservoir solution
Volume of reservoir (μL)	120

### Data collection and processing

The SLA-2*HB01-AS64-sβ_2_m crystal was firstly soaked in reservoir solution supplemented with 17% (*v*/v) glycerol as a cryoprotectant for several seconds and then flash-cooled in a nitrogen stream at − 173 °C [21]. Data collection was carried out using an ADSC Q315 CCD detector at a wavelength of 1.00000 Å. By using beam line BL17U of the Shanghai Synchrotron Radiation Facility (Shanghai, China), the crystal was collected to 2.4 Å resolution. The raw data was indexed, integrated, corrected for absorption, scaled and merged using HKL-2000 [22].

## Results

**Fig. 1:**
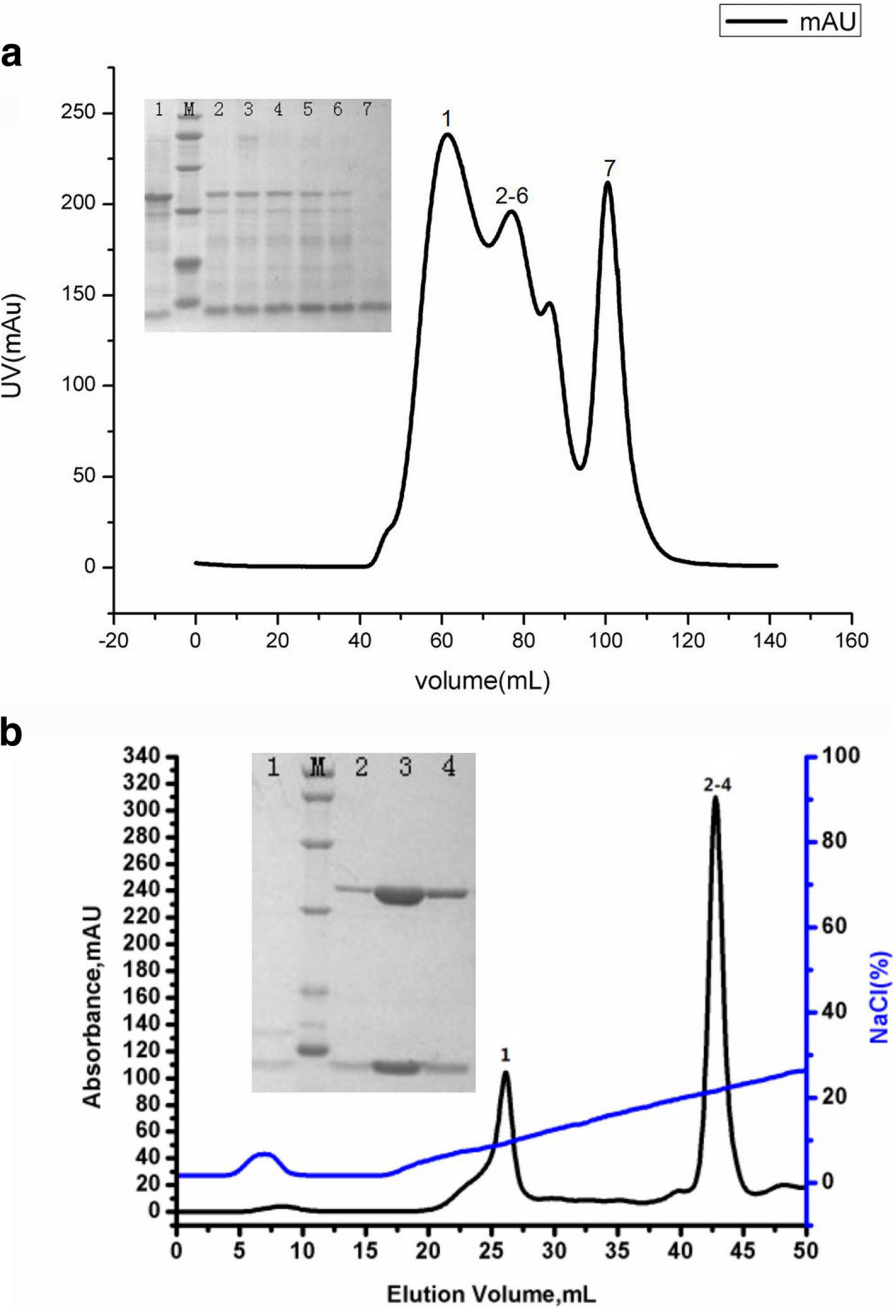
Purification of the refolded SLA-2*HB01-AS64-sβ_2_m by FPLC Superdex 200 16/60 Hi-Load gel-filtration and anion-exchange chromatography Resource Q (GE Healthcare). **a** Gel-filtration profile of the refolded SLA-2*HB01-AS64-sβ_2_m products. Peak 1 represents aggregated heavy chains of SLA-2*HB01, peaks 2–6 represent correctly refolded SLA-2*HB01-AS64-sβ_2_m complex (~ 44 kDa) and peak 7 represents surplus sβ_2_m. Inset: SDS–PAGE gel detection (15%) for peaks 1, 2–6 and 7. M, the low molecular-weight protein ladder (kDa) with 97.1, 66.3, 43.0, 31.0, 20.1 and 14.4. **b** Further purification of the refolded SLA-2*HB01-AS64-sβ_2_m by anion-exchange chromatography Resource Q. Peak 1 contains aggregated light chain of sβ_2_m, peaks 2–4 represent the refolded SLA-2*HB01-AS64-sβ_2_m products, which was eluted with a NaCl concentration of 21.5–26.5%. Inset: SDS-PAGE gel detection (15%) for peaks 1 and 2–4. M, the low molecular-weight protein ladder (kDa) with 97.1, 66.3, 43.0, 31.0, 20.1 and 14.4

After purification to homogeneity by Superdex 200 16/60 HiLoad size-exclusion chromatography, it was shown that the heavy chain of SLA-2*HB01 was successfully refolded together with the light chain of sβ_2_m and the CTL epitope of AS64. The yield of the SLA-2*HB01-AS64-sβ_2_m complex is about 10%. It was shown four peaks in the chromatographic elution profile (Fig. 1a). The peak 1 should be aggregated heavy chains of SLA-2*HB01. The peaks 2–6 detected in lanes 2 to 6 with SDS-PAGE should be the refolded SLA-2*HB01-AS64-sβ_2_m complex (44 kDa). The peak 7 detected in lane 7 with SDS-PAGE should be surplus sβ_2_m (see inset in Fig. 1a). Another peak between peak 2–6 and peak 7, not shown in SDS-PAGE detection, should be some degraded proteins according to our previous practice. The refolded SLA-2*HB01-AS64-sβ_2_m complex was further purified by an anion-exchange chromatography Resource Q with a NaCl elution concentration of 21.5–26.5%. It was shown two peaks for the further purified complex (Fig. 1b). The peak 1 detected in lane 1 with SDS-PAGE contains free sβ_2_m. The peaks 2–4 detected in lanes 2 to 4 with SDS-PAGE contain two bands at the expected molecular weights of SLA-2*HB01 heavy chain (32 kDa) and sβ_2_m light chain (12 kDa) (see inset in Fig. 1b).

**Fig. 2:**
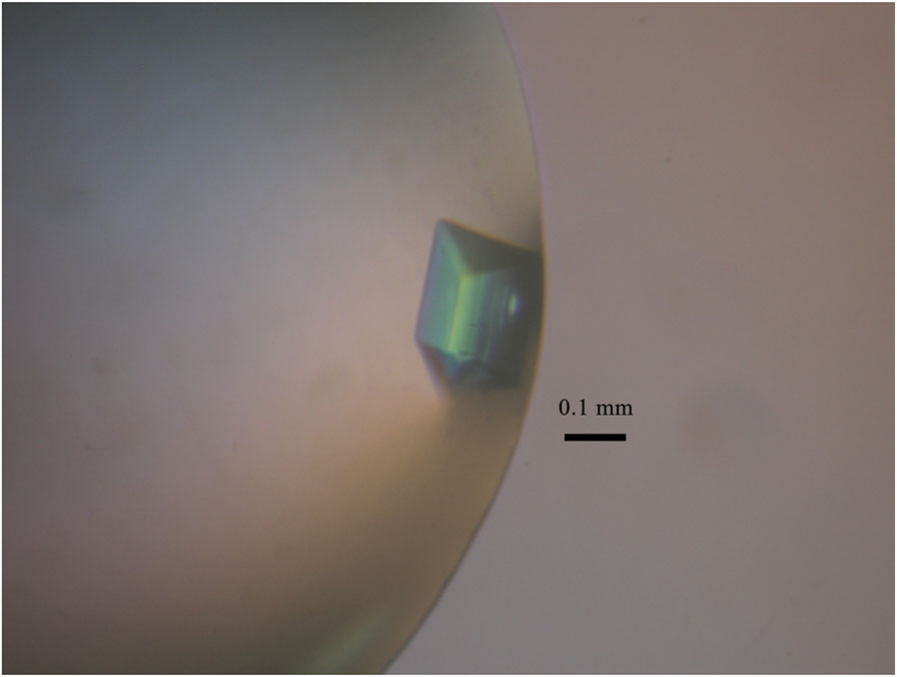
Picture of the SLA-2*HB01-AS64-sβ2m crystal growing in the Index solution No. 79 at 4 °C

**Fig. 3:**
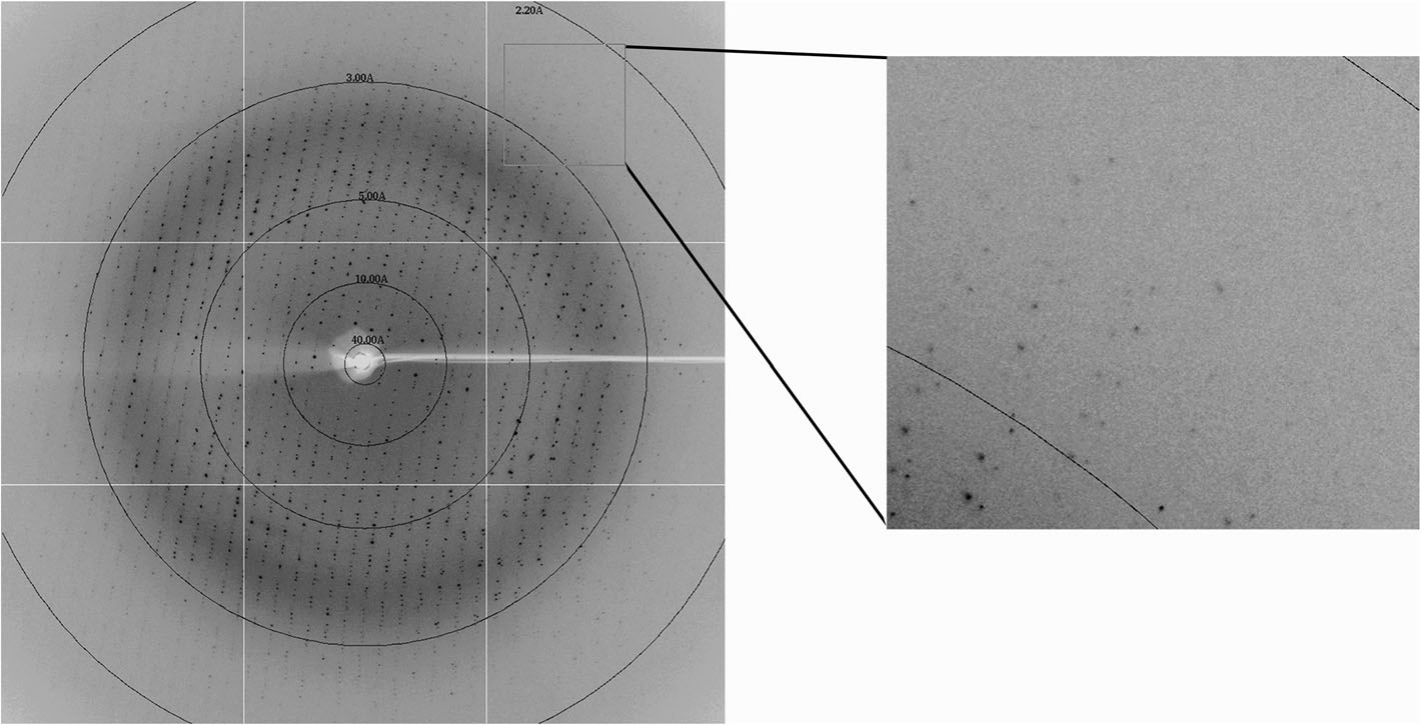
Diffraction pattern of the SLA-2*HB01-AS64-sβ_2_m complex. Spots corresponding to diffraction to high resolution are highlighted in the box

The purified SLA-2*HB01-AS64-sβ_2_m was concentrated to 15 mg/mL for crystal screening. Crystals occurred after 7 days in the Index solution No. 79 at 4 °C and then they were harvested at 14th d (Fig. 2). After preliminary X-ray diffraction analysis, it was shown that the diffraction resolution of the SLA-2*HB01-AS64-sβ_2_m crystal used for data collection was 2.4 Å. The space group of the crystal belongs to P2_1_2_1_2_1_. The unit-cell parameters of the crystal were shown as a = 48.37, b = 97.75, c = 166.163 Å (Fig. 3). The crystal has a solvent content of 61.9%. Data-collection and processing statistics are summarized in Table 3.

**Table 3:** X-ray diffraction data and processing statistics. Values in parentheses are for the highest resolution shell

Diffraction source	BL17U, SSRF
Wavelength (Å)	1.00000
Temperature (°C)	−173
Detector	ADSC Q315 CCD
Crystal-to-detector distance (mm)	350.00
Space group	P2_1_2_1_2_1_
Unit-cell parameters (Å)	a = 48.37, b = 97.75, c = 166.16
Resolution range (Å)	50.0–2.40 (2.40–2.49) ^a^
Total No. of reflections	222,792
No. of unique reflections	31,827
Completeness (%)	99.2
Average *I/σ*(*I*)	21.8
R_merge_^b^ (%)	7.8 (31.8)
Redundancy	7.0 (7.1)

^a^Values in parentheses refer to statistics in the outermost resolution shell

^b^R_merge_ = ∑_hkl_∑_i_ ∣I_i_ -〈I〉∣∑_hkl_∑_i_I_i_, where I_i_ is the observed intensity and 〈I〉 is the average intensity of multiple observations of symmetry related reflections

## Discussion

Previously, we reported a multiple amino-acid sequence alignment between SLA-2*HB01 and other known MHC class I alleles in swine, human and mouse [10]. Based on human HLA-A2 crystal data, the structure of SLA-2*HB01 was predicted by using homology modeling. We noticed SLA-2*HB01 preserved some key functional sites of HLA-A2 and H-2, which indicated that SLA-2*HB01 should be crucial in binding and presenting antigenic peptides [23, 24]. In addition, SLA-2*HB01 has 85.0–93.9, 86.2–97.0 and 83.3–88.6% sequence identity to other SLA-1, SLA-2 and SLA-3 alleles, respectively. Comprehensive analysis of SLA-2*HB01, it should be a novel allele of SLA-2 with specific genetic characteristics [10].

In this work, the crystal of the SLA-2*HB01 molecule complexed with swine β_2_m and a CTL epitope AS64 derived from the Asia1 serotype of FMDV was reported. It seems the space group type of SLA-2*HB01-AS64-sβ_2_m crystal is consistent with that of SLA-1 crystal but quite different from that of SLA-3 crystal, which indicates the structure of SLA-2 might be more similar to that of SLA-1 [17, 18]. Recently, a crystal of SLA-2*HB01 complex associated with an Hu64 CTL epitope derived from O serotype of FMDV was reported. It was shown that the two crystals were similar in space group type, but different in unit-cell parameters [19]. To learn about the elaborate structural characteristics of SLA-2, especially the special characteristics that differ from SLA-1 and SLA-3, the 3-dimentional structure of the SLA-2 complex associated more CTL epitopes derived from swine-origin virus is required to be revealed as soon as possible.

## Conclusions

The research data will be used to further elucidate the 3-dimentional structure of the SLA-2 molecules and design more refined viral epitopes.

## Abbreviations

APCs: Antigen presenting cells
CTL: Cytotoxic T lymphocyte antigen
DMSO: Dimethyl sulfoxide
FMDV: Foot-and-mouth disease virus
IPTG: Isopropyl-β-D-thiogalactopyranoside
LB: Luria–Bertani medium
MHC: Major histocompatibility complex
SLA: Swine leukocyte antigen
